# Smoking, poor nutrition, and sexually transmitted infections associated with pelvic inflammatory disease in remote North Queensland Indigenous communities, 1998-2005

**DOI:** 10.1186/s12905-015-0188-z

**Published:** 2015-04-01

**Authors:** Ming Li, Robyn McDermott

**Affiliations:** School of Population Health, Sansom Institute for Health Research, University of South Australia, Adelaide, Australia; Faculty of Medicine, Health & Molecular Sciences, James Cook University, Cairns, Australia

**Keywords:** Pelvic inflammatory disease, Australian indigenous women, Smoking, Red cell folate, Sexually transmitted infections

## Abstract

**Background:**

Indigenous women in remote North Queensland have a high prevalence of unhealthy lifestyle behaviors and associated health conditions such as sexual transmitted infections (STI). The association of severe pelvic inflammatory disease (PID) with these factors has not been studied. The purpose of this study is to associate the factors with severe PID, as indicated by hospitalization in a high risk population in North Queensland Indigenous communities.

**Methods:**

A cross-sectional association of 1445 Indigenous women using linked hospital separation and survey data during 1998–2005.

**Results:**

The mean age of participating women was 37.4 years, 60% were of Aboriginal and 40% were Torres Strait Island (TSI) people. More than half of them (52.5%) were smokers, 9.3% had chlamydia and 2.6% had gonorrhoea with the overall prevalence of STI among those less than 25 years of age being 23.9%. Among the 47 participants diagnosed with PID in the study period, 42.5% were under 25 years and 95.7% (45 cases) were under 55 years (OR 2.5, 95% CI 1.2-4.1 among women younger than 25 compared to those 25 years and over). PID was strongly associated with smoking (OR 3.1, 95% CI 1.4-9.2) independent of age, ethnicity, STI and folate status. Low red cell folate increased PID hospitalization by 4 times (95% CI 1.5-13.2 of lowest quartile compared to the highest quartile) regardless of age. Having a STI significantly increased the likelihood of severe PID by 2.2 times (95% CI: 1.03-4.5) in Indigenous women younger than 45 years, independent of smoking and folate level. The risk of PID hospitalization was higher for gonorrheal infections (OR 3.2, 955 CI 1.1-9.6) compared to chlamydial infections (OR 1.5 95% CI 0.7-3.5).

**Conclusions:**

Young Indigenous women in North Queensland communities are at very high risk for STI and PID. Smoking, low folate, and STI are clustered, and are associated with PID hospitalizations. Much of this can be prevented with improved nutrition and access to preventive services, especially tobacco control, regular STI screening and treatment, as well as more investment in sexual health education and awareness.

**Electronic supplementary material:**

The online version of this article (doi:10.1186/s12905-015-0188-z) contains supplementary material, which is available to authorized users.

## Background

Pelvic inflammatory disease (PID) is defined as inflammation of the upper genital tract including the endometrium, fallopian tubes and/or contiguous structures that follow infection from micro-organisms that ascend from the cervix and/or vagina [1]. It is associated with long-term morbidity including infertility, chronic pelvic pain, and ectopic pregnancy which are costly to individuals, and health care systems [2], but it is preventable and treatable with early diagnosis and effective management of sexually transmitted infections (STI) [3,4]. It is theorized that factors that impair the immune system, including poor nutrition, may increase susceptibility to bacterial vaginosis (BV), which is associated with nonchlamydial-nongonococcal PID [5,6]. An American cohort study among non-pregnant women found an association between BV and the high dietary intake of folate (OR 0.4, 95% CI: 0.2-0.8) [7]. Factors associated with an increased risk of PID also include young age, lower socioeconomic status, unsafe sexual behaviours, smoking, and contraceptive practice [4,8]. Recent studies show that cardiovascular morbidities such as stroke and atherosclerosis are associated with PID; a variety of mechanisms are involved including immune reaction, endothelial dysfunction, or oxidized low-density lipoprotein [9,10]. Indigenous Australians experience high levels of educational, employment and social disadvantage. The prevalence of smoking or overweight/obesity is twice that for non-Indigenous Australians [11]. The diagnosis rate of gonorrhoea and chlamydia was 20–37 times and 5 times respectively relative to that in non-Indigenous population during 2008–2012 [12]. The hospitalization rate for PID among Aboriginal and Torres Strait Islands (TSI) women in the Northern Territory and North Queensland was 9 times the rate of non-Indigenous women [13,14]. One of the purposes of The Well Person Health Check (WPHC), a screening program conducted in 23 North Queensland Indigenous communities during 1998–2000, was to detect and treat STI and to identify and manage the risk factors for chronic diseases including diabetes, renal and cardiovascular disease. The WPHC found high prevalence and incidence of central obesity and diabetes, poor nutrition, high rates of alcohol use and tobacco smoking together with young maternal age [15], and very high rates of bacterial STI among young people aged 15–24. In particular, the prevalence of chlamydia and gonorrhoea was 23% and 8% respectively in women, and those at highest risk had the poorest access to health services [16]. Among child-bearing Indigenous women participating in the WPHC, the high prevalence of STI and high rates of risky drinking are associated with miscarriage [17]. In spite of the direct and indirect evidence relating PID with STI, folate status, and cardiovascular conditions among various populations using different study designs, the association of these factors with PID has not been explored among Australian Indigenous women. This study documents the population characteristics of PID and identifies behavioral, nutritional, and STI factors associated with PID in both Aboriginal and TSI women, indicated by hospitalization during 1998–2005 in 23 North Queensland Indigenous communities.

## Methods

### Study population

A total of 1445 Indigenous women aged 15 years and over participated in the WPHC were included in this report. The WPHC was a cross-sectional survey conducted in rural and remote Indigenous communities in Far North Queensland during 1998–2000. Methods for this study have been reported in detail elsewhere [16]. Briefly, all residents in 23 communities aged 15 years and over were invited through various media, and word of mouth via the local health service, community council and community groups and 3811 people (participation rate 44.5%) participated. Greater participation rates were observed in the smaller communities. The cohort was demographically representative of the indigenous population of the local area when compared to local population census data. Written informed consent was obtained from participants. The study protocols were approved by the Cairns Base Hospital Human Research Ethics Committee with support from the peak Indigenous health organizations, Apunipima Cape York Health Council and the Torres Strait and Northern Peninsula Area Health Council.

### Anthropometric and biochemical measurements

Participants were asked to remove foot wear and heavy clothing and were weighed to the nearest 0.1 Kg. Height and waist circumference (WC) were recorded to the nearest centimeter with the latter measured by the same technician at the level of the umbilicus. Fruit and vegetable intake was assessed using a methodology derived from that used in the National Nutrition Survey 1995 [18]. Physical activity was self-reported and categorized using the WHO criteria in which ‘enough’ means doing moderate to vigorous physical activity for more than 30 min/day for 5 days in the week before the survey [19]. The consumption of cigarettes and alcoholic drinks were detailed among those who self-reported as current smokers and alcohol drinkers.

Red cell folate (RCF), fasting total cholesterol, HDLC, triglycerides, and glucose were measured from blood samples collected in the early morning (after a minimum 8 hours fast) by a medical officer, registered nurse or trained phlebotomist as described in detail elsewhere [16]. RCF was measured using the Bayer Advia Centaur automated immunoassay system (Bayer, Australia) by Queensland Health Pathology Service in Brisbane with low RCF defined as less than 295 nmol/L. Blood glucose and blood lipids were measured using the photometric enzyme endpoint assay with Cobas Integra 700/400 (Roche Diagnostics, New York, USA). Blood pressure (BP) was the average of three measurements taken sitting after 10 minutes rest.

Polymerase Chain Reaction (PCR) testing (Roche Amplicor CT/NG, Branchburg NJ, USA) for Chlamydia trachomatis and Neisseria gonorrhea was conducted on all urine specimens. Those with a detected STI were recalled for treatment and referred to district sexual health services for contact tracing and follow-up testing.

### Linkage of WPHC data and hospitalizations data

Hospitalization records from public hospitals during 1998–2005 for consenting WPHC participants were identified via a manual search (by a registered nurse with experience working in the region) of the Queensland Health hospital records systems. As there is no unique patient identifier in Queensland, a mapping table, which linked WPHC reference number, hospital facility code and local unit record number, was developed using name, date of birth, and sex, and matched by probability matching method.

### Ascertainment of pelvic inflammatory disease

Hospitalizations were considered to be PID if they contained an International Classification of Diseases, 9th revision, clinical modification (ICD-9-CM) code commencing with 614.0-5 or 614.7-9 inclusive. For hospitalizations coded to the ICD 10th revision (ICD-10), the diagnosis code range N70.0, N70.1, N70.9, N73.0-6, N73.8-9, N74.2-4, N74.8 inclusive were used. Detailed codes and the corresponding conditions are listed in the Additional file 1.

### Statistical analysis

The mean and proportion with 95% confidence interval (95% CI) of anthropometric, biochemical, and behavioral measurements in the study were calculated and compared by PID hospitalization using t-test or chi-square test or the corresponding non-parametric tests after checking the distributions of the measurements. The prevalence ratio of behavioural and biochemical measurements with PID was calculated using logistic regression and further adjusted for age. Step-wise models were built by including candidate factors with P < =0.3 from univariate regression in the first model and eliminating one each step until only significant factors remained in the final model [20]. The eligible candidate factors included in the regression analysis were age, BMI, hypertension, RCF, STI, and smoking. Only significant factors were presented in Table 1. The strength of smoking, low RCF, and STI was studied in a full model with age, RCF and smoking, and combined STI included. The interaction term between the significant factors was also tested in the model. Subgroup analysis of association between STI and PID among women of productive age (<=45 years) by ethnicity was further conducted. The analysis was conducted using STATA 12 (STATAcorp, College Station, Texas, USA). Statistical significant level was set as P < 0.05.

**Table 1:** **Characteristics of participants by PID status among Indigenous women in Far North Queensland during 1998-2005**

	**No PID N = 1398**	**Yes N = 47**	**Overall N = 1445**
	**Mean or % (95% CI)**	**Mean or % (95% CI)**	**Mean or % (95%CI)**
Mean age (years)*	37.6 (36.8-38.5)	28.9 (25.8-32.0)	37.4 (36.6-38.2)
**Age groups %**			
15-24	91.6 (87.3-94.6)	8.4 (5.4-12.7)	24.3 (22.1-26.6)
25-34	93.8 (89.9-96.2)	6.2 (3.8-10.1)	25.5 (23.4-27.9)
35-54	96.6 (93.8-98.2)	3.4 (1.8-6.2)	34.0 (31.6-36.5)
> = 55	98.7 (94.8-99.7)	1.3 (0.3-5.2)	16.2 (14.4-18.2)
**Aboriginal %**	60.7 (58.1-63.2)	68.1 (54.6-81.6)	60.9 (58.4-63.4)
**Height (cm)**	161.0 (160.6-161.3)	159.9 (158.1-161.7)	160.9 (160.6-161.2)
Weight (KG)*	74.4 (73.3-75.5)	66.7 (59.8-73.7)	74.2 (73.1-75.2)
WC (cm)*	96.6 (95.7-97.6)	88.9 (83.7-94.0)	96.4 (95.5-97.3)
**BMI (Kg/m** ^**2**^ )*	28.7 (28.2-29.1)	25.9 (23.5-28.3)	28.6 (28.2-29.0)
**BMI categories %**			
<18.5	9.2 (7.5-11.3)	17.0 (8.7-30.7)	8.7 (7.4-10.3)
18.5-24.9	26.1 (23.3-29.1)	34.0 (21.9-48.7)	26.7 (24.5-29.1)
> = 25	64.7 (61.5-67.8)	48.9 (34.9-63.1)	64.6 (62.0-67.0)
Systolic BP (mmHg)*	127.1 (125.9-128.2)	118.4 (115.1-121.8)	126.8 (125.7-127.9)
**Diastolic BP (mmHg)**	68.8 (68.1-69.5)	67.5 (64.4-70.6)	68.8 (68.1-69.4)
Hypertension %*	30.6 (27.6-33.7)	6.4 (2.0-18.2)	28.8 (26.5-31.2)
Total Cholesterol (mmol/L)*	4.78 (4.73-4.83)	4.39 (4.04-4.73)	4.8 (4.7-4.9)
**Triglycerides (mmol/L)**	1.58 (1.52-1.64)	1.52 (1.20-1.84)	1.58 (1.52-1.64)
LDL (mmol/L)*	2.93 (2.89-2.98)	2.59 (2.33-2.84)	2.9 (2.87-3.0)
**HDL (mmol/L)**	1.14 (1.13-1.16)	1.12 (1.03-1.20)	1.14 (1.12-1.16)
**Hyperlipidemia %**	0.7 (0.2-1.4)	0	0.5 (0.2-1.0)
**Blood glucose (g/L)**	5.8 (5.6-5.9)	5.3 (4.5-6.0)	5.8 (5.6-5.9)
**Diabetes %**	17.3 (14.9-19.9)	12.8 (5.8-25.8)	15.8 (14.1-17.8)
RCF (nmol/L)*	436.5 (427.1-445.9)	360.6 (319.4-401.8)	434.0 (424.8-443.2)
RCF quintile*			
1st IQR 562-716	25.2 (22.4-28.2)	8.7 (3.3-21.2)	25.0 (22.8-27.4)
2nd IQR 437-490	25.6 (22.7-28.6)	19.6 (10.4-33.7)	25.0 (22.8-27.4)
3rd IQR 340-392	23.3 (20.6-26.3)	34.8 (22.4-49.6)	25.0 (22.8-27.4)
4th IQR 208-286	25.9 (23.1-29.0)	37.0 (24.2-51.8)	25.0 (22.8-27.4)
Smoking %*	51.6 (49.0-54.2)	80.9 (69.5-92.2)	52.5 (50.0-55.1)
**Median number of cigarettes (IQR)**	12 (5–20)	10 (5–15)	12 (5–20)
**Drinking %**	58.6 (56.0-61.2)	70.2 (57.0-83.4)	59.0 (56.4-61.6)
**PA enough %**	20.7 (18.5-22.8)	17.0 (6.1-27.9)	20.6 (18.5-22.6)
**Fruit > =2 and/or vegetable > =5 serves/day %**	1.6 (1.0-2.3)	0	1.6 (0.9-2.2)
**Chlamydia %**	9.1 (7.6-10.6)	14.9 (4.6-25.2)	9.3 (7.8-10.8)
Gonorrhea %*	2.4 (1.6-3.2)	8.5 (0.4-16.6)	2.6 (1.7-3.4)
Chlamydia/gonorrhea %*	9.9 (7.9-11.9)	21.3 (9.4-33.1)	10.5 (8.8-12.0)

*P < 0.05 using t-test or chi-square test; RCF quartile IQR: the interquartile range of red cell folate.

*Hypertension defined as SBP > =140 and/or DBP > =90 mmHg; Hyperlipidemia defined as cholesterol > 5.5 or triglycerides > 2.0 or HDL > 2.0 mmol/L; Diabetes was defined as either clinical diagnosis verified by the participants’ medical records or a 2 hour glucose tolerance test, or fasting blood glucose level > 7.0 mmol/L.

*Physical activity enough defined as meeting the WHO recommendation of more than 30 min per day doing moderate to vigorous physical activity for 5 days in the week prior to the survey [19].

*Chlamydia and gonorrhea detected using PCR for Chlamydia trachomatis and Neisseria gonorrhea on all urine specimens.

## Results

There were 1445 eligible female participants aged 15–89 (mean age 37.4 years) included in this study. Of them, 60% were of Aboriginal and 40% were TSI people. 52.5% were smokers and 20.6% reported “sufficient” physical activity. Only 1.6% participants reported having more than 2 serves of fruits and 5 serves of vegetables daily and one in five had RCF less than 295 nmol/L. The detected rates of chlamydia and gonorrhea were 9.3% and 2.6% respectively with a combined STI rate of 10.5%. Among the total 149 participants with STI, 84 women were aged less than 25 years and 31 were aged 25–35 years (20.8%) with 134 cases of women less than 45 years.

Forty seven women had PID recorded in the diagnosis fields in hospitalization records with a prevalence of 5.1% (95% CI: 3.8-6.7). Of them, 8 cases were dated 8–31 months before the survey, and the rest were 1 month to 7 years after survey. Among the 47 PID patients, 42.3% were aged 15–24 years, 31.9% were aged 25–34 years, and 25.5% were aged over 35 years. PID prevalence did not vary significantly by ethnicity. 80.9% of the PID patients were smokers and none reported having more than 2 serves of fruit and/or 5 serves of vegetables daily. More than one fifth (21.2%) of the PID patients had baseline chlamydia and/or gonorrhea infection. The PID patients had lower BMI, systolic BP, cholesterol, LDL, RCF concentration but higher detected rates of STI compared to those without PID. PID prevalence was not significantly different by diabetes, hyperlipidemia, physical activity level and drinking status (Table 1).

Those aged 15–24 and 25–34 years were 7 times (95% CI: 1.6-30.3) and 5 times (95% CI: 1.1-21.7) respectively more likely being hospitalized for PID compared to women aged over 55 years. Using over 35 years as a comparison reference, the likelihood ratio of being hospitalized as PID patients aged 25–34 and 15–24 years was 2.5 (95% CI: 1.2-5.4) and 3.6 times (95% CI: 1.7-7.4). Those diagnosed with chlamydia and/or gonorrhea infection were 2.5 times (95% CI: 1.2-5.1) more likely to be hospitalized with PID and this association was attenuated to null after adjustment for age. Smokers and those with lower baseline RCF levels had 3 times the likelihood of being hospitalized with PID compared to their corresponding counterparts regardless of age. Smoking attenuated the association between low RCF and PID to null (OR 2.8, 95% CI: 0.9-8.5 compared to the highest quartile) (Table 2).

**Table 2:** **Factors associated with PID among Indigenous women in Far north Queensland 1998-2005**

	**Crude OR (95% CI)**	**Age Adjusted OR**	**Full model**
**RCF quartile (reference: 1st quartile of RCF IQR 562–716)**			
2nd IQR 437-490	2.3 (0.7-7.5)	2.0 (0.6-6.6)	1.7 (0.6-5.8)
3rd IQR 340-392	4.1 (1.4-12.5)	3.4 (1.1-10.5)	2.8 (0.9-8.7)
4th IQR 208-286	4.4 (1.5-13.2)	3.5 (1.2-10.7)	2.8 (0.9-8.5)
**Smoking (reference: No)**			
Yes	4.0 (1.9-8.3)	3.1 (1.5-6.6)	2.8 (1.3-6.0)
Chlamydia* (reference: No)			
Yes	1.8 (0.8-4.0)	1.2 (0.5-2.7)	
Gonorrhea* (reference: No)			
Yes	3.8 (1.3-11.3)	2.4 (0.8-7.2)	
**Chlamydia/gonorrhea (reference: No)**			
Yes	2.5 (1.2-5.1)	1.7 (0.8-3.5)	

*Chlamydia and gonorrhea detected using PCR for Chlamydia trachomatis and Neisseria gonorrhea on all urine specimens.

Table 3 shows the PID prevalence odds ratio by chlamydia and gonorrhea among women of productive age by ethnicity. The overall pattern of the association among this subgroup was consistent with that among the overall population in this study. STI increased the risk of PID hospitalization by 2.2 times (95% CI 1.03-4.5). Gonorrhoea (OR 3.2, 95% CI 1.1-9.6) was stronger than chlamydia (OR 1.5 95% CI 0.7-3.5) in the association with PID. Aboriginal women had significantly higher risk of PID hospitalization if infected with chlamydia/gonorrhea (OR 2.8, 95% CI 1.2-6.4) than TSI women (OR 1.5, 95% CI 0.3-7.2).

**Table 3:** **Odds ratio (95% CI) of chlamydia/gonorrhea with PID among Indigenous women of productive age (<=45 years) in Far North Queensland during 1998–2005**

	**Aboriginal**	**TSI**	**overall**
Chlamydia* (reference: No)			
Yes	2.1 (0.8-5.4)	0.7 (0.1-5.7)	1.5 (0.7-3.5)
Gonorrhea* (reference: No)			
Yes	3.4 (1.0-12.4)	3.4 (0.4-29.6)	3.2 (1.1-9.6)
**Chlamydia/gonorrhea (reference: No)**			
Yes	2.8 (1.2-6.4)	1.5 (0.3-7.2)	2.2 (1.03-4.5)

*Chlamydia and gonorrhea detected using PCR for Chlamydia trachomatis and Neisseria gonorrhea on all urine specimens.

Excluding the 8 PID cases dated before the survey, the analysis does not change the association of PID hospitalization between RCF, smoking, and STI among the study population, either in the overall cohort or in the cohort of reproductive age.

## Discussion

In summary, we found very high rates of severe and preventable PID in this cohort of women, where PID accounted for 5.1% of all hospitalizations over the period 1998–2005. Teenage girls and young women to 24 years had the highest rates of PID, which is consistent with other studies [4,21]. The hospitalization data would substantially underestimate the true prevalence of PID in this population, as less severe disease is not generally reported centrally. PID can be diagnosed and treated in the primary care setting if it is detected early to avoid complications and hospitalization [22]. Australian data estimates over 59,000 PID encounters annually in general practice of which only 0.3% results in hospital referral [23]. High prevalence of PID has been reported elsewhere among Aboriginal women and vastly underdiagnosed and poorly treated [24]. Diagnosis is clinician dependent and an awareness of PID as a possible differential diagnosis and a high index of suspicion is necessary. Even in areas specializing in sexual health medicine, diagnostic rates for PID differ significantly between clinicians resulting in cases being missed. There are no clear diagnostic criteria and laparoscopy remains the gold standard despite the cost, invasive nature and lack of both specificity and sensitivity [25]. Undiagnosed or inadequately treated PID leads to poorer reproductive health outcomes in the long term. Increased awareness of PID symptoms, diagnosis and treatment and a revision of the guidelines are needed to improve detection and management of PID in this high risk setting [24].

Our study found that low RCF, and smoking were associated with severe PID independent of age. Folate, as an indicator of the general nutritional quality of the diet, is an essential micronutrient found in a range of foods but highest in green leafy vegetables, citrus fruit, nuts, lentils, and liver. Low serum folate is associated with impaired T cell and neutrophil function, and deficiency of folate is associated with an increased risk of bacteriuria [26]. Increased folate may improve immunity and reduce the risk of BV [7], which is associated with non STI PID [5,6]. Further exploration of the relationship between BV and vaginal flora and PID in this population is needed to close the knowledge gap. We have reported smoking was associated with low RCF, especially among young Indigenous women of childbearing age in Far North Queensland [27], which can explain our observation in the current study about the mediation effect of smoking between RCF and PID. Smoking was the strongest factor associated with PID independent of age, RCF and STI. This is consistent with US case–control studies, which found odds ratios of 1.7-2.3 for smoking after adjustment for age, number of recent sexual partners, frequency of intercourse, and previous episodes of gonorrhea [28,29]. Plausible mechanisms of association between smoking and PID include the impairment of immune response to infection and estrogen [4,30-32], ovum transport and tubal ciliary function to repel effectively ascending infectious organisms [33].

It is worth noting that chlamydia and gonorrhea were associated with PID in this analysis, especially in Aboriginal women but not in TSI women. This could be due to the relatively small numbers in the TSI cohort, although there were a relatively higher number of PID events; or that patients with STI PID in Aboriginal communities had better primary health referral or easier access to public hospital than those in the TSI communities. Further investigation of primary health services for STI PID patients in these communities could help to answer the ethnic differences in the association between STI and PID. Among Indigenous women of reproductive age, we found gonorrhea was strongly than chlamydia associated with PID hospitalization. This is consistent with reports of an incident rate ratio of 3.5 (95% CI 2.5-4.8) and the same pattern of PID association with gonorrhea and chlamydia in a follow up study among 38193 Australian women of the same age group during 2000–2008 [34]. One of the weaknesses of our study is the lack of information about sexual behaviors and contraceptive practice, and also the cross-sectional nature of the baseline survey, which would not ascertain subsequent bacterial infection and the temporal relation with PID in the study population. Further information about PID reported from the patients and primary health care services is needed to thoroughly describe PID occurrence among this study population and its association with STI. Other studies in this population show extremely low awareness of and knowledge about STIs and HIV [35]. Subsequent to our study, the “Young Persons Check” which includes PCR screening and treatment for bacterial STIs has been implemented in some communities, however this needs to be augmented by sustained and effective community awareness programs.

## Conclusions

Young Australian Indigenous women in North Queensland are at high risk of sexual transmitted infections. Hospitalization for PID was highest among younger women aged 15–25 years. STI, lower RCF and smoking were strongly associated with hospitalization for PID.

## Additional file

Additional file 1:
**ICD codes and pelvic inflammatory disease conditions.** Listed in the file are the ICD codes used to generate pelvic inflammatory disease from matched hospitalization separations in the study population as described in the paper. It includes both ICD-9 and ICD-10 versions with corresponding conditions.

## Abbreviations

PID: pelvic inflammatory disease
STI: sexually transmitted infections
WPHC: The Well Person Health Check
WC: waist circumference
RCF: red cell folate
BP: blood pressure
PCR: Polymerase Chain Reaction
ICD codes: International Classification of Diseases codes
TSI: Torres Strait Islands
