# Supplementary material for: Smoking, poor nutrition, and sexually transmitted infections associated with pelvic inflammatory disease in remote North Queensland Indigenous communities, 1998-2005
Source: BMC Womens Health. 2015 Apr 1;15:31. doi: 10.1186/s12905-015-0188-z (PMC4392641; doi:10.1186/s12905-015-0188-z)
Supplement: Additional file 1: — ICD codes and pelvic inflammatory disease conditions. Listed in the file are the ICD codes used to generate pelvic inflammatory disease from matched hospitalization separations in the study population as described in the paper. It includes both ICD-9 and ICD-10 versions with corresponding conditions. [file 12905_2015_188_MOESM1_ESM.doc]

**Additional file 1: ICD codes and pelvic inflammatory disease conditions**

ICD-9 code

- 614.0 Acute salpingitis and oophoritis
- 614.1 Chronic salpingitis and oophoritis
- 614.2 Salpingitis and oophoritis not specified as acute, subacute, or chronic
- 614.3 Acute parametritis and pelvic cellulitis
- 614.4 Chronic or unspecified parametritis and pelvic cellulitis
- 614.5 Acute or unspecified pelvic peritonitis, female
- 614.7 Other chronic pelvic peritonitis, female
- 614.8 Other specified inflammatory disease of female pelvic organs and tissues
- 614.9 Unspecified inflammatory disease of female pelvic organs and tissues

ICD -10 codes:

- N70 Salpingitis and oophoritis
- N70.0 Acute salpingitis and oophoritis
- N70.1 Chronic salpingitis and oophoritis Incl.: Hydrosalpinx
- N70.9 Salpingitis and oophoritis, unspecified
- N73 Other female pelvic inflammatory diseases
- N73.0 Acute parametritis and pelvic cellulitis

Incl.:

• Abscess of:

o broad ligament

o parametrium

• Pelvic cellulitis, female • specified as acute

- N73.1 Chronic parametritis and pelvic cellulitis

Incl.: Any condition in N73.0 specified as chronic

- N73.2 Unspecified parametritis and pelvic cellulitis

Incl.: Any condition in N73.0 unspecified whether acute or chronic

- N73.3 Female acute pelvic peritonitis
- N73.4 Female chronic pelvic peritonitis
- N73.5 Female pelvic peritonitis, unspecified
- N73.6 Female pelvic peritoneal adhesions

Excl.: postprocedural pelvic peritoneal adhesions (N99.4)

- N73.8 Other specified female pelvic inflammatory diseases
- N73.9 Female pelvic inflammatory disease, unspecified

Incl.: Female pelvic infection or inflammation NOS

- N74* Female pelvic inflammatory disorders in diseases classified elsewhere
- N74.2* Female syphilitic pelvic inflammatory disease
- N74.3* Female gonococcal pelvic inflammatory disease
- N74.4* Female chlamydial pelvic inflammatory disease
- N74.8* Female pelvic inflammatory disorders in other diseases classified elsewhere
